# Calycosin inhibits breast cancer cell migration and invasion by suppressing EMT via BATF/TGF-β1

**DOI:** 10.18632/aging.203093

**Published:** 2021-06-07

**Authors:** Zhenxia Zhang, Min Lin, Junli Wang, Fenglian Yang, Peikui Yang, Yaqun Liu, Zikai Chen, Yuzhong Zheng

**Affiliations:** 1 School of Life Sciences and Food Engineering, Hanshan Normal University, Chaozhou 521041, Guangdong, China; 2 Center of Reproductive Medicine, Affiliated Hospital of Youjiang Medical University for Nationalities, Baise 533000, Guangxi, China; 3 School of Pharmacy, Youjiang Medical University for Nationalities, Baise 533000, Guangxi, China

**Keywords:** breast cancer, calycosin, BATF, TGFβ1, invasion and metastasis

## Abstract

In this study, we investigated the effects of calycosin on breast cancer cell progression and their underlying mechanisms. Calycosin dose- and time-dependently inhibited proliferation, migration, and invasion by T47D and MCF-7 breast cancer cells by downregulating basic leucine zipper ATF-like transcription factor (BATF) expression. Moreover, BATF promoted breast cancer cell migration and invasiveness by increasing TGFβ1 mRNA and protein levels. Bioinformatics analysis, dual luciferase reporter assays, and chromatin immunoprecipitation assays confirmed the presence of BATF-binding sites in the promoter sequence of *TGFβ1* gene. Calycosin treatment inhibited epithelial-mesenchymal transition (EMT) of breast cancer cells by significantly increasing E-cadherin levels and decreasing N-cadherin, Vimentin, CD147, MMP-2, and MMP-9 levels through downregulation of BATF and TGFβ1. TGFβ1 knockdown reduced the migration and invasiveness of BATF-overexpressing breast cancer cells, whereas incubation with TGFβ1 enhanced the migration and invasiveness of calycosin-treated breast cancer cells. Our findings demonstrated that calycosin inhibited EMT and progression of breast cancer cells by suppressing BATF/TGFβ1 signaling. This suggests calycosin would be a promising therapeutic option for breast cancer patients.

## INTRODUCTION

Breast cancer is the most common cause of cancer-related deaths among women worldwide [1]. The rates of breast cancer incidence have been increasing constantly in the last few decades [2]. In China, approximately 1.6 million new cases of breast cancer were reported in 2020 [3]. Improvements in diagnosis and therapeutic strategies have increased the survival rates among breast cancer patients in recent decades, but the prognosis of advanced stage breast cancer patients remains poor because of the high rates of drug resistance and disease recurrence [4, 5]. Furthermore, the efficacy of current treatments in breast cancer patients with distant metastasis is poor. Therefore, there is an urgent need for new and effective treatment strategies against breast cancer metastasis.

The incidence rates of breast cancer are significantly lower in East Asians countries compared to individuals from USA and Europe [6]. This is partly attributed to higher dietary consumption of phytoestrogens by East Asians. Phytoestrogens are plant-derived non-steroidal compounds that are structurally similar to 17β-estradiol and demonstrate estrogenic effects on breast cancer cells [7]. Plant-derived phytoestrogens are sub-divided into four main classes—isoflavones, coumestans, lignans, and stilbenes. Soy food consumption is associated with lower breast cancer incidence, disease recurrence, and mortality because it contains high levels of isoflavones [8, 9].

Calycosin is a natural isoflavone isolated from *Astragali radix* with anti-oxidative, anti-inflammatory and anti-cancer properties [10]. Calycosin inhibits invasion and metastasis of colorectal cancer cells by suppressing epithelial-to-mesenchymal transition (EMT) [11]. Calycosin inhibited *in vitro* growth of pancreatic cancer cells by inducing cell cycle arrest and apoptosis; however, it also induced metastatic progression of pancreatic cancer in an orthotopic tumor xenograft mouse model by modulating the tumor microenvironment [12]. The role of calycosin in breast cancer is not known.

The basic leucine zipper ATF-like transcription factor (BATF) family includes three members—BATF1, BATF2 and BATF3 [13]. BATFs belong to the AP-1/ATF super-family of transcription factors and act as inhibitors of AP-1 activity [14]. AP-1 family of transcription factors regulates cellular proliferation, differentiation, and survival by activating or downregulating the expression of their target genes [15, 16]. Several studies have reported the regulatory role of BATFs in cancer [17, 18]. For example, BATF inhibits epithelial-mesenchymal transition (EMT) in colorectal cancer cells by downregulating TGF-β. However, the functional role of BATFs in breast cancer is poorly understood. Hence, in this study, we investigated the effects of calycosin on breast cancer cell progression and the underlying mechanisms, including its effect on BATF expression and functions in breast cancer cells.

## RESULTS

### Calycosin inhibits *in vitro* proliferation, migration and invasiveness of breast cancer cells

**Figure 1:**
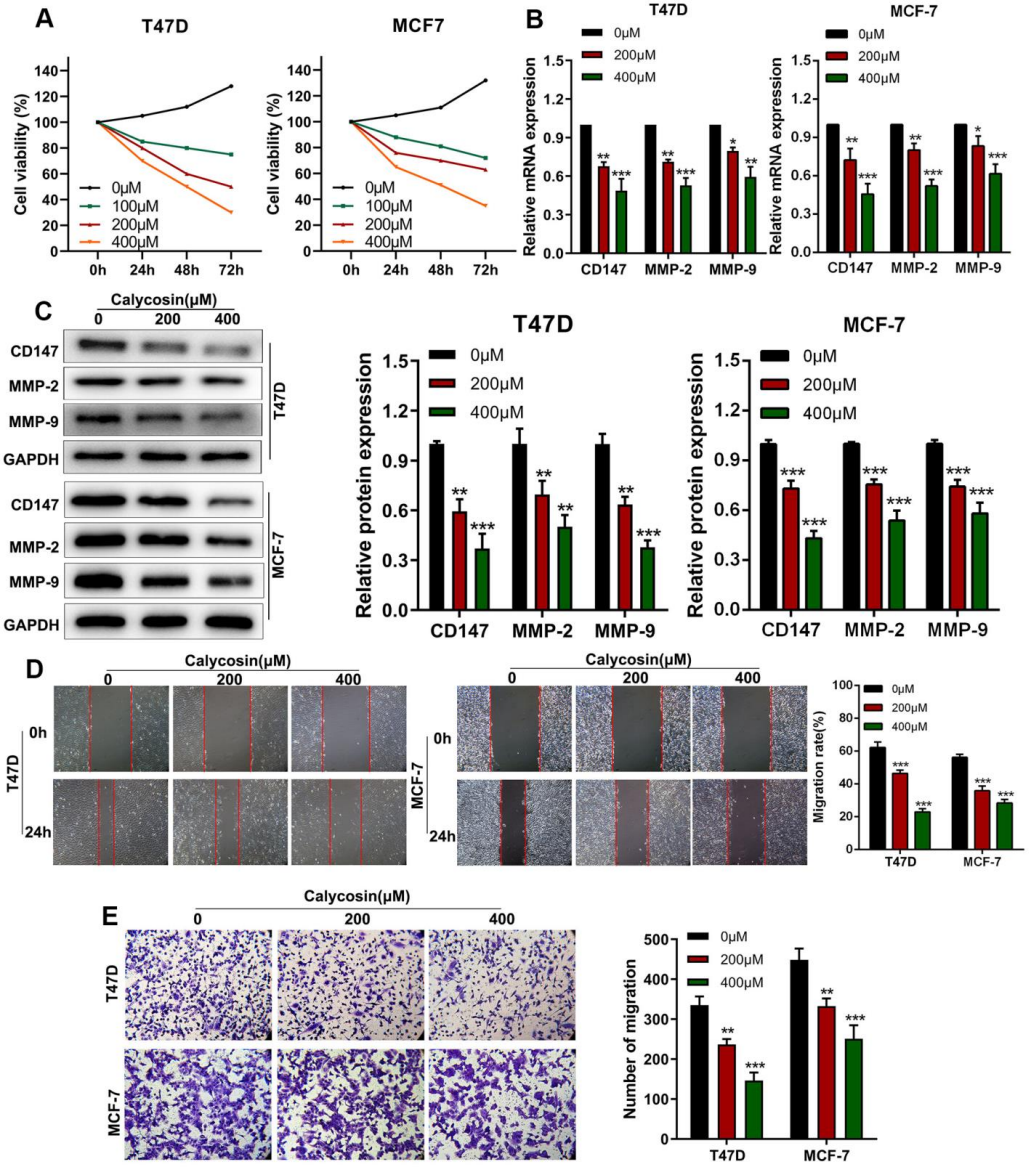
**Calycosin inhibits *in vitro* invasion and migration of breast cancer cells in a dose-dependent manner.** (**A**) CCK-8 assay results show proliferation rates of control or calycosin-treated T47D and MCF-7 cells at 0, 24, 48, and 72 h. (**B**) RT-qPCR analysis shows relative levels of CD147, MMP-2, and MMP-9 transcripts in control or calycosin-treated T47D and MCF-7 cells. (**C**) Western blot analysis shows expression levels of CD147, MMP-2, and MMP-9 proteins in control or calycosin-treated T47D and MCF-7 cells. (**D**) Representative images show wound healing assay results in control or calycosin-treated T47D and MCF-7 cells at 0 h and 24 h. (**E**) Transwell invasion assay results show invasiveness of control or calycosin-treated T47D and MCF-7 cells. The data were represented as means ± SD. *P<0.05; **P<0.01; ***P<0.001.

CCK-8 assay showed that calycosin inhibited proliferation of T47D and MCF-7 breast cancer cells in a dose- and time-dependent manner (Figure 1A). Previous studies have shown that higher expression of CD147, MMP-2, and MMP-9 is associated with breast cancer progression [19, 20]. RT-qPCR and western blot results showed that calycosin treatment downregulated the mRNA and protein levels of CD147, MMP-2, and MMP-9 in T47D and MCF-7 breast cancer cells (Figure 1B, 1C). Wound healing and Transwell invasion assay results showed that calycosin treatment decreased migration and invasion rates of breast cancer cells, respectively, in a dose-dependent manner (Figure 1D, 1E). These results showed that calycosin inhibited *in vitro* breast cancer cell progression in a dose-dependent manner.

### Calycosin inhibits breast cancer cell progression by suppressing BATF expression

**Figure 2:**
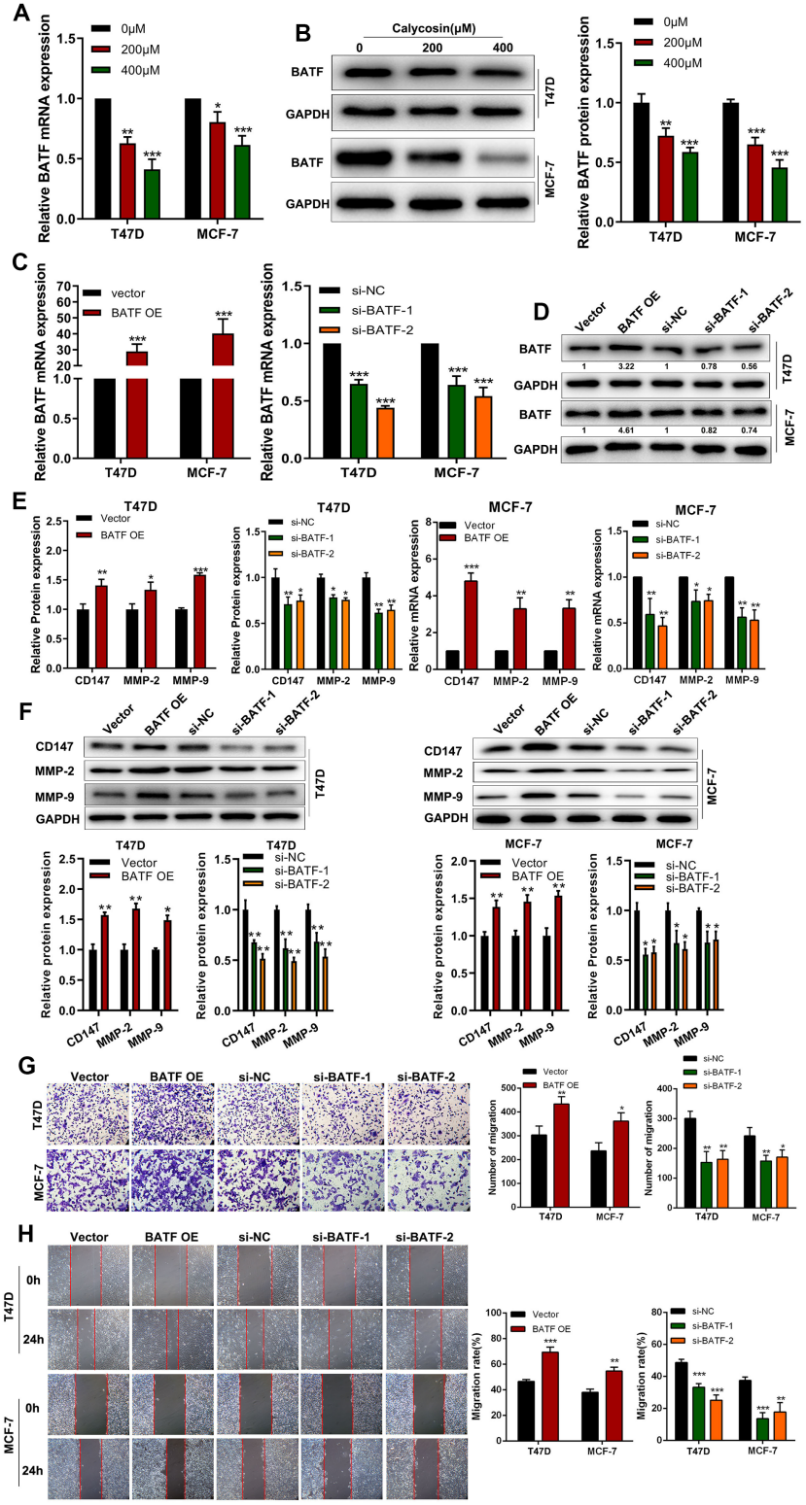
**Calycosin treatment reduces BATF levels in breast cancer cells.** (**A**) RT-qPCR analysis shows relative levels of BATF mRNA in T47D and MCF-7 cells treated with different concentrations of calycosin. (**B**) Western blot analysis shows the relative levels of BATF protein in T47D and MCF-7 cells treated with different concentrations of calycosin. (**C**, **D**) RT-qPCR and western blot analyses show the relative levels of BATF mRNA and protein in control, BATF-overexpressing, and BATF-silenced T47D and MCF-7 cells. (**E**) RT-qPCR analysis shows relative levels of CD147, MMP-2, and MMP-9 transcripts in control, BATF-overexpressing, and BATF-silenced T47D and MCF-7 cells. (**F**) Western blot analysis shows relative levels of CD147, MMP-2, and MMP-9 proteins in control, BATF-overexpressing, and BATF-silenced T47D and MCF-7 cells. (**G**) Transwell invasion assay results show invasiveness of control, BATF-overexpressing, and BATF-knockdown T47D and MCF-7 cells. (**H**) Wound healing assay results show migration rates of control, BATF-overexpressing, and BATF-knockdown T47D and MCF-7 cells. The data were presented as means ± SD. *P<0.05; **P<0.01; ***P<0.001.

RT-qPCR and western blot analyses showed that calycosin treatment downregulated BATF mRNA and protein levels in T47D and MCF-7 breast cancer cells (Figure 2A, 2B). Therefore, we analyzed the effects of BATF overexpression or knockdown on *in vitro* breast cancer cell migration and invasiveness. BATF mRNA and protein levels were significantly upregulated in BATF-overexpressing T47D and MCF-7 breast cancer cells and downregulated in si-BATF-transfected T47D and MCF-7 breast cancer cells (Figure 2C, 2D). The levels of CD147, MMP-2 and MMP-9 transcripts and proteins were significantly increased in BATF-overexpressing breast cancer cells and significantly reduced in BATF-silenced breast cancer cells (Figure 2E, 2F). Wound healing and Transwell invasion assays showed that breast cancer cell migration and invasiveness was significantly increased by BATF overexpression and significantly reduced by BATF silencing (Figure 2G, 2H). These results demonstrated that BATF overexpression induced breast cancer cell progression.

### Calycosin counteracts pro-metastatic effects of BATF in breast cancer cells

**Figure 3:**
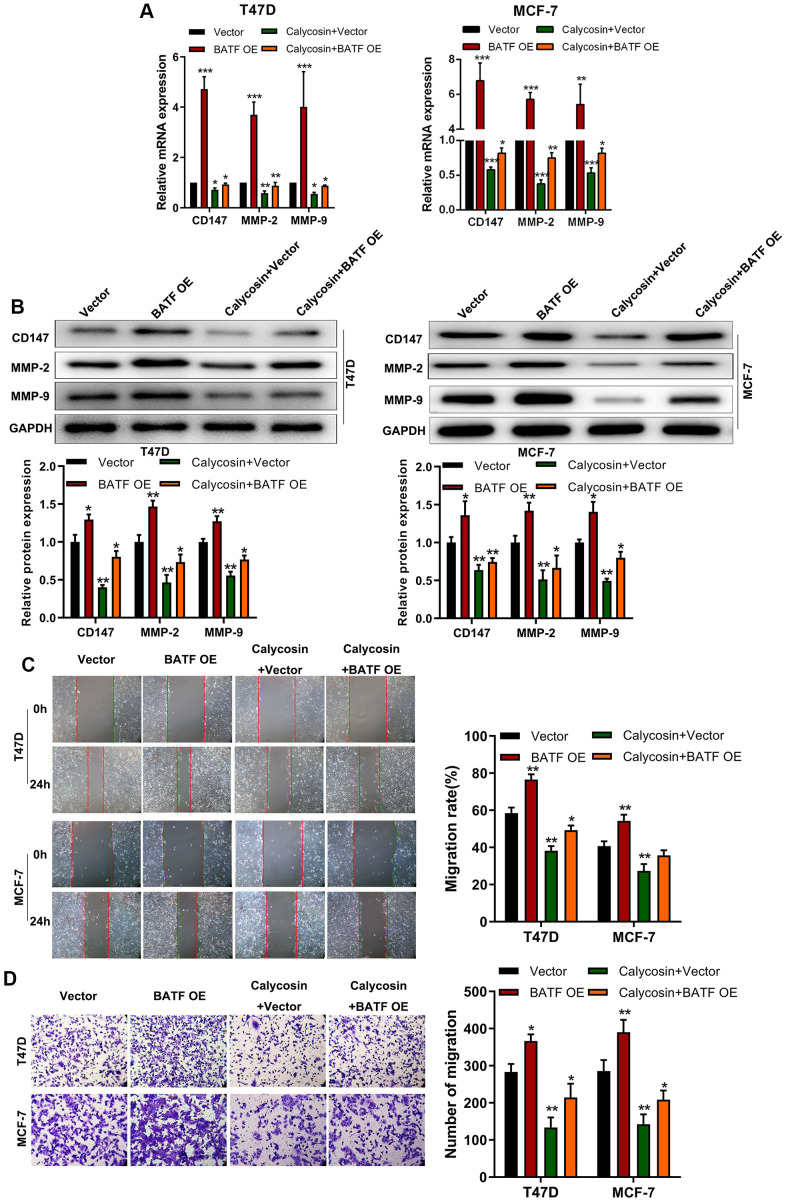
**Calycosin counteracts pro-metastatic effects of BATF in breast cancer cells.** (**A**) RT-qPCR analysis shows relative levels of CD147, MMP-2, and MMP-9 transcripts in control and calycosin-treated BATF-overexpressing T47D and MCF-7 cells. (**B**) Western blot analysis shows relative levels of CD147, MMP-2, and MMP-9 proteins in control and calycosin-treated BATF-overexpressing T47D and MCF-7 cells. (**C**) Representative images show the migration ability of control and calycosin-treated BATF-overexpressing T47D and MCF-7 cells at 0 and 24 h. (**D**) Transwell invasion assay results show invasiveness of control and calycosin-treated BATF-overexpressing T47D and MCF-7 cells. The data were represented as means ± SD. *P<0.05; **P<0.01; ***P<0.001.

We then analyzed if calycosin suppressed invasion and migration of BATF-overexpressing breast cancer cells. Calycosin treatment decreased the expression of CD147, MMP-2, and MMP-9 transcripts and proteins in BATF-overexpressing breast cancer cells (Figure 3A, 3B). Furthermore, calycosin treatment significantly reduced migration and invasiveness of BATF-overexpressing breast cancer cells (Figure 3C, 3D). These results showed that calycosin inhibited pro-metastatic effects of BATF in breast cancer cells.

### Calycosin downregulates expression of TGFβ1 by suppressing BATF

**Figure 4:**
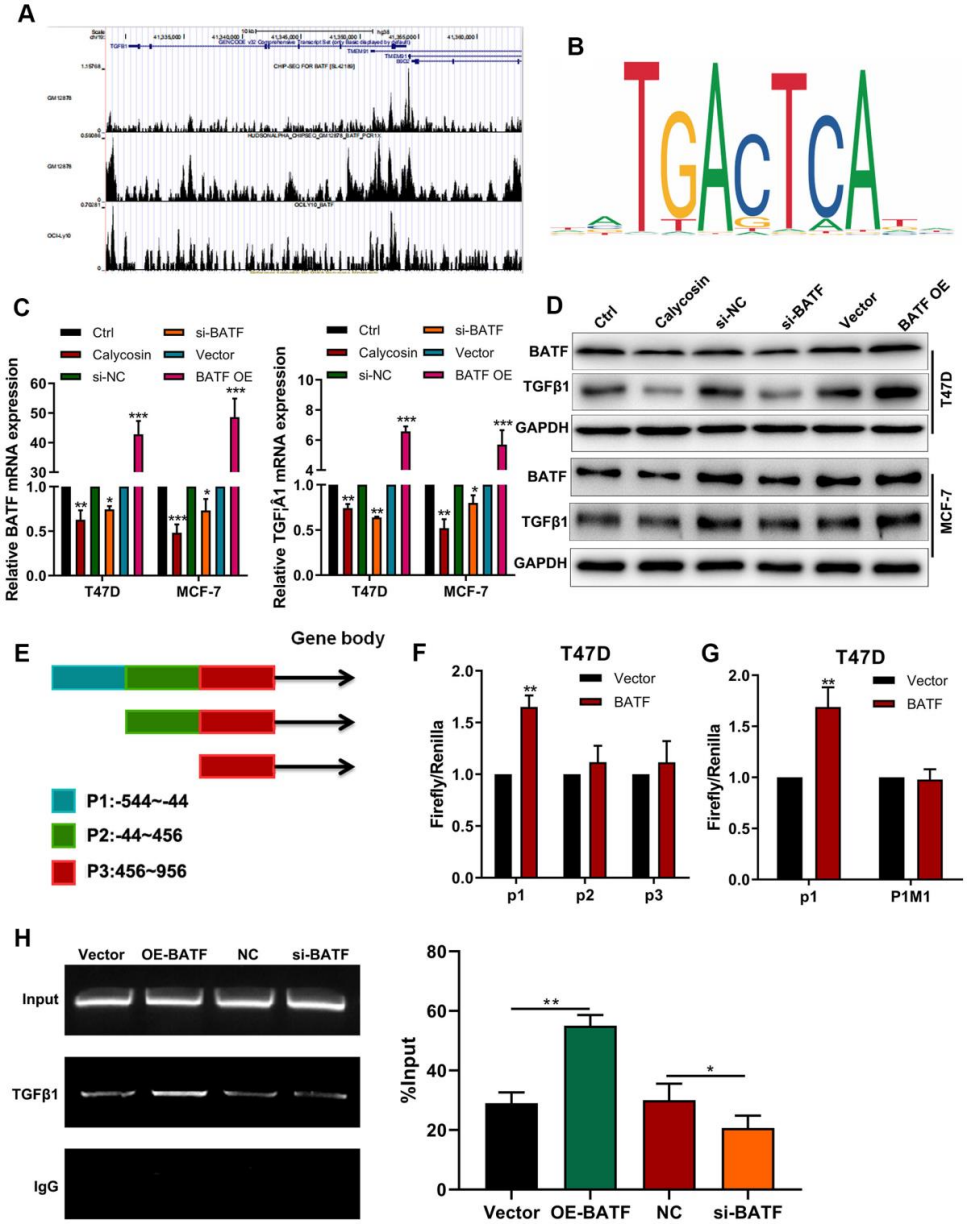
**Calycosin downregulates TGFβ1 expression via BATF.** (**A**) ChIP-seq database analysis shows potential BATF binding sites in the *TGFβ1* gene. (**B**) JASPAR database analysis shows potential BATF binding motif. (**C**) RT-qPCR analysis shows relative levels of BATF and TGFβ1 transcripts in calycosin-treated BATF-overexpressing, and BATF-knockdown T47D and MCF-7 cells and corresponding controls. (**D**) Western blot analysis shows relative levels of BATF and TGF*β*1 proteins in control and calycosin-treated BATF-overexpressing and BATF-knockdown T47D and MCF-7 cells. (**E**) JASPAR database analysis shows 3 potential BATF binding sites in the promoter sequence of *TGFβ1* gene (P1: -544~-44, P2: -44~456, P3: 456~956). (**F**) Dual-luciferase reporter assay results show relative luciferase activity in breast cancer cells transfected with luciferase reporter vectors containing P1, P2, or P3 promoter sequence of *TGFβ1* gene. (**G**) Dual-luciferase reporter assay results show relative luciferase activity in breast cancer cells transfected with luciferase reporter vectors containing either wild-type P1 (P1 WT) or mutated P1 (P1 MUT) promoter sequence of *TGFβ1* gene. (**H**) ChIP-PCR assay results confirm direct binding of BATF to *TGFβ1*. The data were represented as means ± SD. *P<0.05; **P<0.01; ***P<0.001.

ChIP-seq online database [21] analysis potential BATF binding sites in *TGFβ1* (Figure 4A). We also identified the binding motif of BATF using the JASPAR database (Figure 4B). Calycosin treatment significantly downregulated the levels of BATF and TGFβ1 transcripts and proteins in breast cancer cells (Figure 4C, 4D). Furthermore, TGFβ1 mRNA and protein levels were significantly increased by BATF overexpression and significantly reduced by BATF knockdown in breast cancer cells (Figure 4C, 4D). Next, we identified 3 potential BATF-binding sequences in the *TGFβ1* promoter sequence (P1: -544~-44, P2: -44~456, P3: 456~956) using the JASPAR database (Figure 4E). We cloned P1, P2, or P3 sequences of the *TGFβ1* gene promoter into luciferase reporter vectors and transfected the recombinant vectors into breast cancer cells. Dual luciferase reporter assay results showed that BATF significantly increased luciferase activity in cells transfected with luciferase reporter vector containing P1, but luciferase activity was not observed in cells transfected with luciferase reporter vectors containing P2 or P3 (Figure 4E). This suggested potential BATF-binding sites in the P1 sequence of the *TGFβ1* promoter. We then constructed recombinant luciferase reporter vectors containing either wild-type or mutated P1 sequence of *TGFβ1* (P1WT or P1M1). Dual luciferase reporter assay results showed that luciferase activity was significantly higher in cells transfected with luciferase reporter vector containing wild type P1 sequence, but luciferase activity was not observed in cells transfected with the luciferase reporter vector containing mutated P1 sequence (P1MUT) (Figure 4F, 4G). These results confirmed that BATF binds to the P1 sequence of the *TGFβ1* promoter. We also performed ChIP-PCR to confirm direct binding between BATF and TGFβ1. ChIP-PCR results showed that *TGFβ1* levels were significantly reduced in the BATF-knockdown T47D cells and significantly increased in the BATF-overexpressing T47D cells (Figure 4H). These results confirmed that calycosin downregulated the expression of TGFβ1 by suppressing BATF.

### Calycosin suppresses breast cancer cell progression by inhibiting EMT

**Figure 5:**
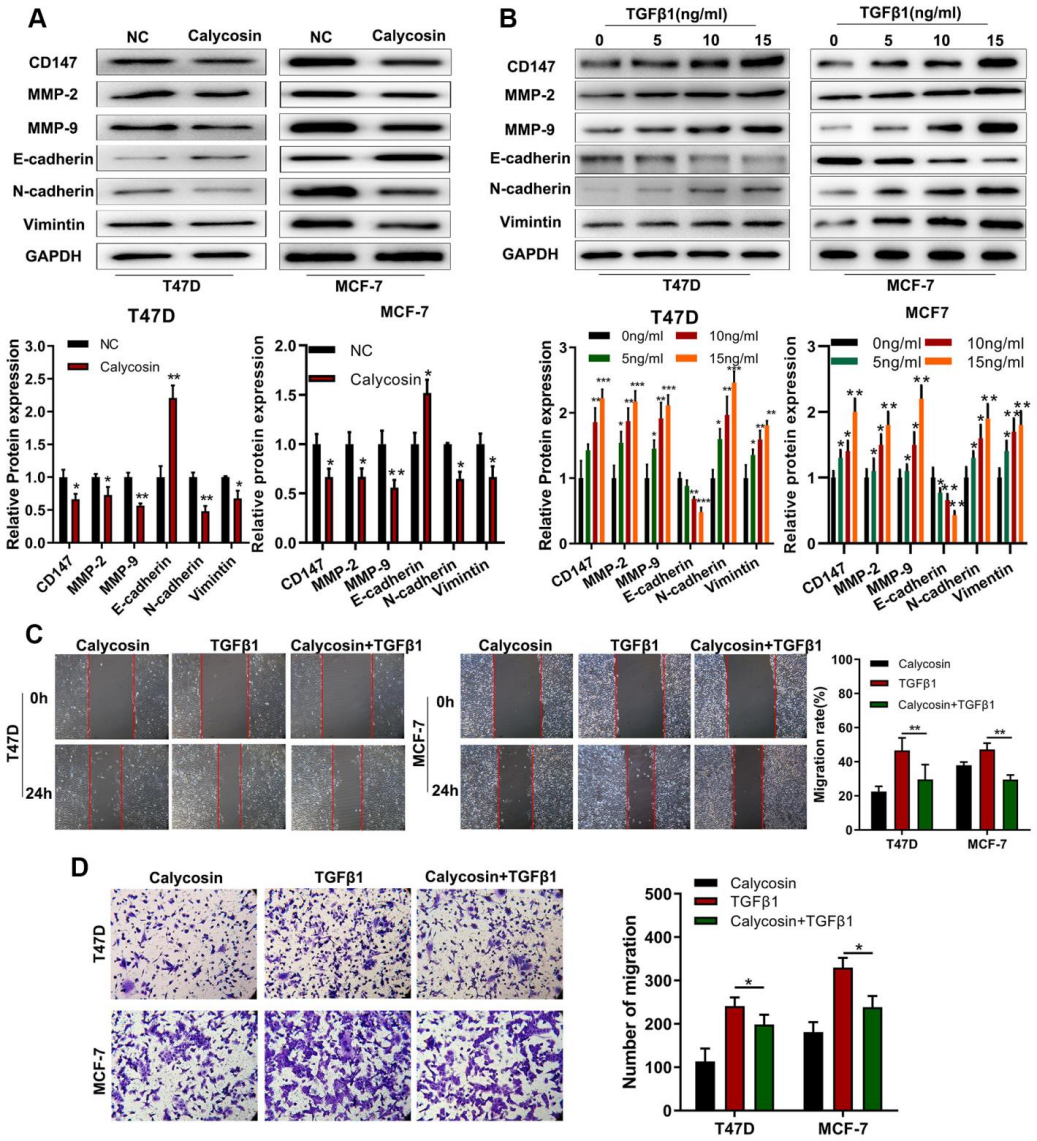
**Calycosin suppresses breast cancer cell migration and invasiveness by inhibiting EMT.** (**A**) Western blot analysis shows relative levels of CD147, MMP-2, MMP-9, E-cadherin, N-cadherin, and Vimentin proteins in control and calycosin-treated T47D and MCF-7 breast cancer cells. (**B**) Western blot analysis shows relative levels of CD147, MMP-2, MMP-9, E-cadherin, N-cadherin, and Vimentin proteins in T47D and MCF-7 breast cancer cells treated with different concentrations of TGFβ1. (**C**) Representative images of wound healing assay at 0 and 24 h show migration ability of T47D and MCF-7 breast cancer cells treated with calycosin, TGFβ1, or calycosin plus TGFβ1. (**D**) Transwell invasion assay results show the invasiveness of T47D and MCF-7 breast cancer cells treated with calycosin, TGFβ1, or calycosin plus TGFβ1. The data were represented as means ± SD. *P<0.05; **P<0.01; ***P<0.001.

TGFβ1 is a crucial regulator of epithelial-mesenchymal transition (EMT), a key process in tumor metastasis [22]. Hence, we investigated the effects of calycosin on EMT in breast cancer cells. Calycosin significantly increased the expression of E-cadherin (epithelial cell biomarker) and decreased the expression levels N-cadherin and Vimentin (mesenchymal cell biomarkers) as well as CD147, MMP-2, and MMP-9 (pro-metastatic proteins) in the breast cancer cells (Figure 5A). Conversely, TGFβ1 treatment decreased E-cadherin levels and increased N-cadherin, Vimentin, CD147, MMP2 and MMP9 expression levels in the breast cancer cells (Figure 5B). TGFβ1 treatment also significantly increased the migration and invasiveness of breast cancer cells, but calycosin reduced the migration and invasiveness of TGFβ1-treated breast cancer cells (Figure 5C, 5D). These results showed that calycosin suppressed migration and invasiveness of breast cancer cells by inhibiting EMT via BATF/ TGFβ1.

### Calycosin inhibits breast cancer cell progression by suppressing BATF/ TGFβ1 axis

**Figure 6:**
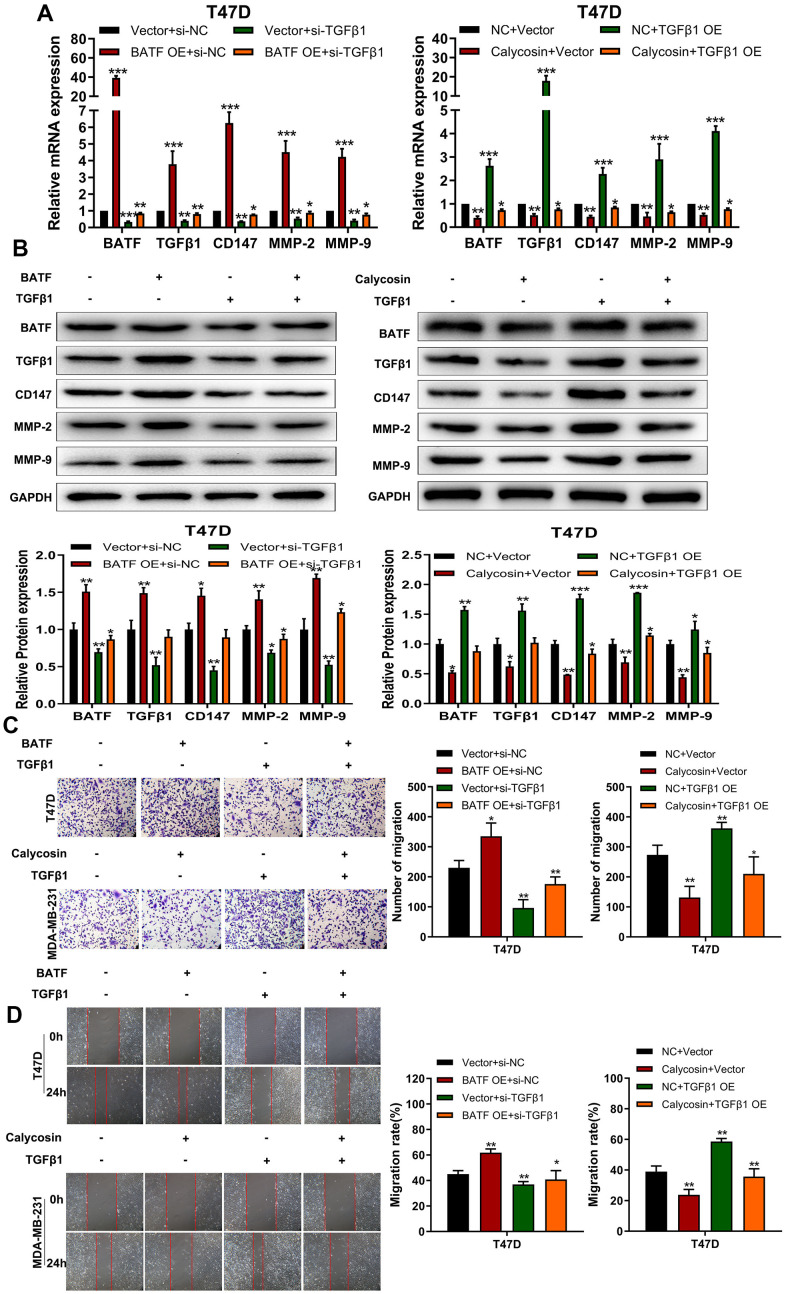
**Calycosin inhibits breast cancer cell migration and invasiveness via BATF/TGFβ1.** (**A**) RT-qPCR analysis shows relative levels of BATF, TGFβ1, CD147, MMP-2, and MMP-9 transcripts in BATF-overexpressing cells with or without TGFβ1 knockdown (left panel), and TGFβ1-overexpressing cells treated with or without calycosin (right panel). (**B**) Western blot analysis shows relative levels of BATF, TGFβ1, CD147, MMP-2, and MMP-9 proteins in BATF-overexpressing cells with or without TGFβ1 knockdown (left panel) and TGFβ1-overexpressing cells treated with or without calycosin (right panel). (**C**) Transwell invasion assay results show invasiveness of BATF-overexpressing cells with or without TGFβ1 knockdown (left panel) and TGFβ1-overexpressing cells treated with or without calycosin (right panel). (**D**) Wound healing assay results show the migration ability of BATF-overexpressing cells with or without TGFβ1 knockdown (left panel) and TGFβ1-overexpressing cells treated with or without calycosin (right panel). The data were represented as means ± SD. *P<0.05; **P<0.01; ***P<0.001.

**Figure 7:**
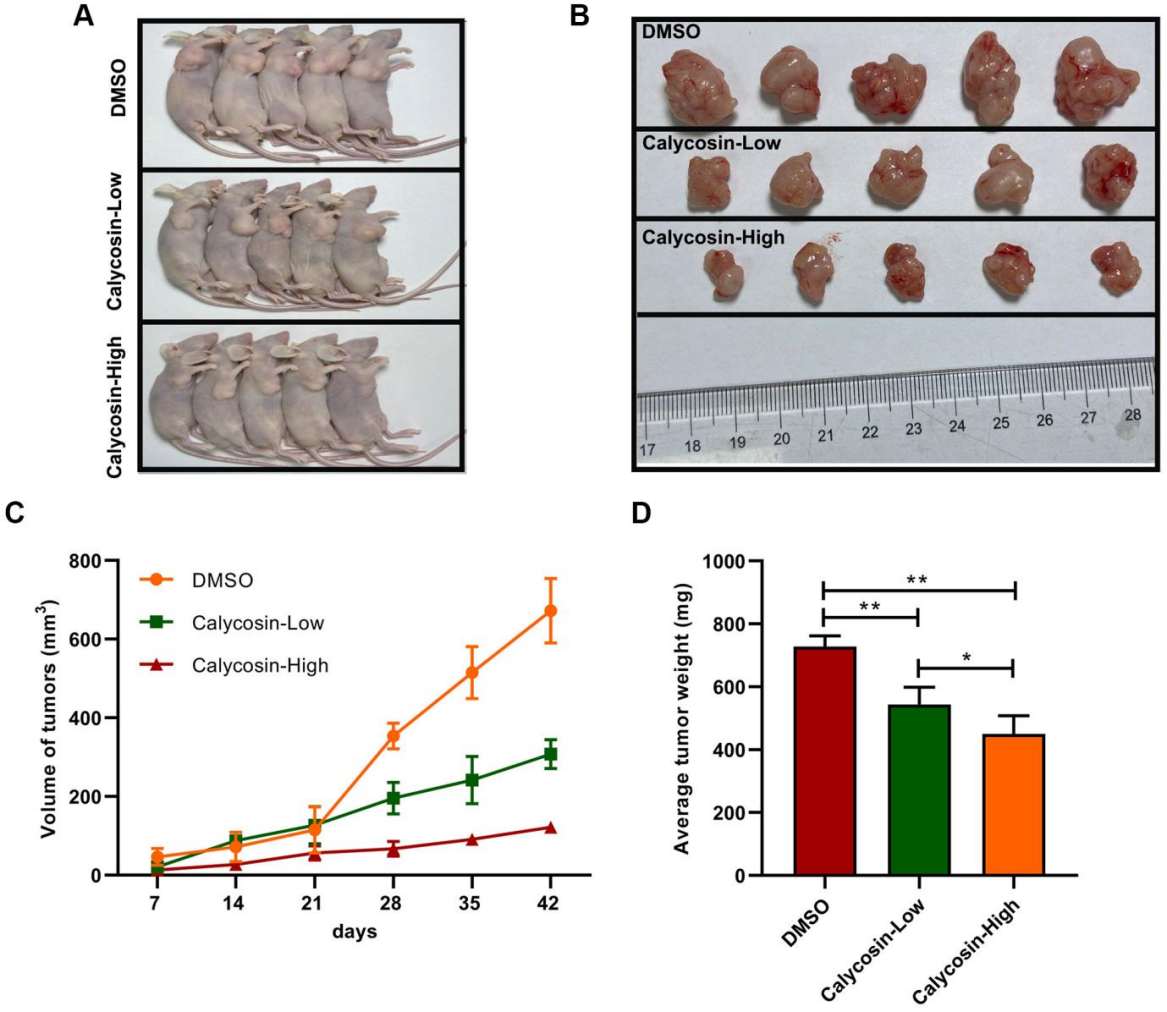
**Calycosin inhibited breast cancer cells growth *in vivo*.** (**A**, **B**) Calycosin inhibited subcutaneous tumorigenesis using nude mics models. (**C**) Tumors volume curves over time. (**D**) The average weight of tumors. The data were represented as means ± SD. *P<0.05; **P<0.01.

The above findings confirmed that BATF/TGFβ1 axis positively regulated the expression of pro-metastatic proteins, CD147, MMP-2, and MMP-9, in breast cancer cells. Therefore, we further investigated the relationship between BATF and TGFβ1. We observed that overexpression of BATF upregulated TGFβ1 mRNA and protein levels in breast cancer cells, whereas, TGFβ1 knockdown decreased BATF mRNA and protein levels in BATF-overexpressing breast cancer cells (Figure 6A, 6B). Furthermore, calycosin treatment significantly reduced the levels of CD147, MMP-2, and MMP-9 in TGFβ1-overexpressing breast cancer cells (Figure 6A, 6B). Transwell invasion assay results showed that TGFβ1 knockdown significantly reduced the invasiveness of BATF-overexpressing breast cancer cells (Figure 6C). However, overexpression of TGFβ1 significantly increased the invasiveness of calycosin-treated breast cancer cells (Figure 6C). Wound healing assay results showed that TGFβ1 knockdown significantly reduced the migration of BATF-overexpressing breast cancer cells compared to the corresponding controls (Figure 6D). However, overexpression of TGFβ1 significantly increased the migration of calycosin-treated breast cancer cells (Figure 6D). In addition, to further determine the effect of Calycosin inhibits breast cancer cells growth *in vivo*, nude mice subcutaneous tumor mode was constructed (Figure 7A). The results revealed that Calycosin in T47D cells markedly decreased the growth rate of subcutaneous xenograft tumors (Figure 7B–7D). In summary, our data demonstrated that calycosin inhibited *in vitro* migration, invasiveness and growth of breast cancer cells by suppressing BATF/ TGFβ1.

## DISCUSSION

Breast cancer is a leading cause of mortality and morbidity among women worldwide, especially in patients with metastatic and recurrent disease [21]. This is in large part because breast cancer is a highly aggressive and often metastatic tumor [23]. Calycosin is a natural isoflavone isolated from *Astragali radix*, which exhibits anti-inflammatory, anti-oxidative and anti-tumor properties [10, 24]. Li et al reported that calycosin inhibited migration and invasion of human breast cancer cells by down-regulating Foxp3 expression [2]. Moreover, calycosin inhibited osteosarcoma cell migration and invasion [25]. Calycosin also inhibited *in vitro* growth and progression of gastric, colorectal, ovarian, and breast cancer cells [26–29]. In this study, we demonstrated that calycosin inhibited proliferation and progression of breast cancer cells in a dose- and time-dependent manner. Calycosin treatment downregulated expression levels of EMT- and metastasis-related proteins such as N-cadherin, Vimentin, CD147, MMP-2, and MMP-9 levels in breast cancer cells. Wound healing and Transwell invasion assays demonstrated that calycosin treatment significantly reduced migration and invasion of breast cancer cells in a dose-dependent manner. This suggested that calycosin inhibited breast cancer progression by suppressing EMT.

Tumor metastasis is significantly associated with EMT, a process that involves cancer cells acquiring mesenchymal characteristics (expression of N-cadherin and vimentin) and losing epithelial features (expression of E-cadherin) [30]. Our results demonstrated that TGFβ1 treatment increased *in vitro* migration and invasiveness of breast cancer cells by inducing EMT and increasing the expression of pro-metastatic proteins such as CD147 [31], MMP2 [32], and MMP9 [33]. Previous studies reported that TGFβ1 promoted EMT in cancer cells [34]. ChIP-seq analysis showed that BATF directly regulated transcription of TGFβ1. Moreover, ChIP-PCR and dual luciferase reporter assays confirmed direct binding of BATF to the promoter region of TGFβ1 (Figure 4).

BATF is a basic leucine zipper nuclear protein belonging to the AP-1/ATF super-family of proteins [35]. BATF is a negative regulator of AP-1-mediated transcription and inhibits cellular transformation by Ras and Fos [36]. In this study, we demonstrated that calycosin reduced BATF protein levels in breast cancer cells in a concentration-dependent manner (Figure 2A–2C). Moreover, BATF overexpression increased migration and invasiveness of breast cancer cells (Figures 2F–2H, 3C, 3D).

In summary, our results demonstrated that calycosin inhibited breast cancer cell progression by suppressing EMT via BATF/TGFβ1. Therefore, calycosin is a promising candidate for breast cancer therapy.

## MATERIALS AND METHODS

### Cell culture

The human breast cancer cell lines, T47D and MCF-7, were purchased from Shanghai Cell Bank of the Chinese Academy of Science (Shanghai, China), and cultured in RPMI-1640 medium (Gibco, CA, USA) supplemented with 10% FBS (Gibco, CA, USA) and 1% Penicillin-Streptomycin solution (Gibco, CA, USA) in a humidified chamber maintained at 37° C and 5% CO_2_. The cells were treated with different concentrations of calycosin and TGFβ1 (Sigma Aldrich, St. Louis, MO, USA).

### SiRNA-mediated knockdown of BATFs and TGFβ1

We purchased the following siRNAs (GenePharma, Shanghai, China) that specifically target BATF-1, BATF-2, and TGFβ1:

si-BATF-1 sense: 5’-GCAGAAGAGUAUUAAGAAAGA-3’,

si-BATF-1 antisense: 5’-UUUCUUAAUACUCUUCUGCAU-3’;

si-BATF-2 sense: 5’-GGCCCAAUGCAGAAGAGUAUU-3’,

si-BATF-2 antisense: 5’-UACUCUUCUGCAUUGGGCCUG-3’;

si-TGFβ1 sense: 5’-GCAUAUAUAUGUUCUUCAACA-3’,

si-TGFβ1 antisense: 5’-UUGAAGAACAUAUAUAUGCUG-3’.

We used scrambled siRNAs as negative controls. We used the siRNAs with highest silencing effect for further studies.

### CCK-8 cell proliferation assay

We seeded breast cancer cells (3000 cells per well) in 96-well plates at 37° C and 5% CO_2_ for 0, 24, 48, and 72 h. Then, the cells were incubated with 10% CCK-8 (Dojindo; Kumamoto, Japan) in normal culture medium for 4 h. Subsequently, we measured optical density at 450 nm in a plate reader according to manufacturer’s instructions.

### Real-time quantitative PCR (RT-qPCR)

We extracted total RNA from T47D and MCF-7 breast cancer cells using TRIzol® reagent (Invitrogen, Carlsbad, CA, USA). We assessed quantity and quality of RNA samples using A260/A280 ratio in a microplate reader. Then, we performed cDNA synthesis using 100 ng of total RNA per sample followed by qPCR using specific primers as listed in Table 1 and Maxima SYBR Green/ROX qPCR Master Mix (Thermo Fisher Scientific, Waltham, MA, USA). The relative expression levels of specific mRNAs were calculated using 2^−∆∆Ct^ method.

**Table 1:** The primer sequences for RT-qPCR.

**Gene**	**Primer**
CD147	Forward: ACTCCTCACCTGCTCCTTGA Reverse: GTCTCCCCCTCGTTGATGTG
MMP-2	Forward: TGGATGATGCCTTTGCTCGT Reverse: TCGGCGTTCCCATACTTCAC
MMP-9	Forward: TCTATGGTCCTCGCCCTGAA Reverse: CATCGTCCACCGGACTCAAA
BATF	Forward: CGTATTGCCGCCCAGAAGAG Reverse: TCTGTTTCTCCAGGTCTTCGC
TGFβ1	Forward: CTGCAAGTGGACATCAACGG Reverse: AAGTTGGCATGGTAGCCCTT
GAPDH	Forward: GGAGCGAGATCCCTCCAAAAT Reverse: GGCTGTTGTCATACTTCTCATGG

### Western blotting

The cells were lysed using RIPA buffer (08714-04, Nacalai Tesque Inc., Kyoto, Japan) and the protein concentration of samples was quantified using BCA assay. Equal amounts of proteins (120 μg) were separated by SDS-PAGE and the separated proteins were transferred onto polyvinylidene difluoride (PVDF) membranes. The membranes were blocked with 5% skimmed milk for 30 mins, and then incubated overnight at 4° C with primary antibodies against BATF (ab236876, dilution 1:1000; Abcam, Cambridge, MA, USA), TGFβ1 (ab92486, 1:1000; Abcam, Cambridge, MA, USA), CD147 (ab188190, dilution 1:5000; Abcam, Cambridge, MA, USA), MMP-2 (ab110258, dilution 1:1000; Abcam), MMP-9 (ab97779, dilution 1:1000; Abcam, Cambridge, MA, USA), and GAPDH (5174S, dilution 1:2000; Cell Signaling Technology, Inc. Danvers, CO, USA). Then, the blots were incubated with horseradish peroxidase-conjugated secondary antibodies (ab6789 or ab6721, dilution 1:500, Abcam) for 30 min at room temperature. The blots were developed and visualized using ECL Plus Western Blotting Detection kit (GE Healthcare, Buckinghamshire, UK).

### Dual luciferase reporter assay

The TGFβ1 mRNA sequence containing BATF binding site was amplified using PCR Amplification kit (TaKaRa Biotechnology, Dalian, China). We generated three recombinant luciferase reporter vectors with P1, P2, or P3 promoter sequences of *TGFβ1* with potential BATF-binding domains. We also generated a recombinant luciferase reporter vector containing mutated P1 promoter sequence of *TGFβ1* gene (P1 MUT). We measured relative luciferase activity using the Dual-Luciferase Reporter assay system (Promega Corporation, Madison, WI, USA) according to manufacturer's protocol.

### Wound healing assay

The breast cancer cells were grown to 70%-80% confluence. Then, the monolayer was scratched with a pipette tip and further cultured for 24 h. The width of the scratch was measured at 0 and 24 h to determine the migration rates of different experimental groups of breast cancer cells.

### Transwell invasion assay

We seeded T47D or MCF-7 cells (1 × 10^4^ cells/well) in 150 μL serum-free DMEM medium into the upper chamber of Transwell chambers coated with matrigel (BD Biosciences, USA). We then added 700 μL of DMEM medium with 10% FBS into the lower chamber. The transwell plates were incubated at 37° C for 24 h. The cells invading the matrigel were fixed with cold methanol, stained with crystal violet, and counted under a light microscope to determine the number of invading cells in each experimental group.

### ChIP-PCR

Chromatin immunoprecipitation experiments were performed using ChIP-IT Express kit (Active Motif) according to manufacturer’s instructions. Briefly, breast cancer cells were incubated in fixing solution containing 1% formaldehyde and protease inhibitor cocktail (PIC1; 1 μg/mL leupeptin, 1.4 μg/mL pepstatin, 0.2 mg/mL PMSF (Sigma), 1 mM EGTA, and 1 mM EDTA) for 10 min with gentle rotation. The cross-linking reaction was quenched by adding Glycine Stop-Fix solution. Then, the cells were pelleted, resuspended in ice-cold lysis buffer, and dounce-homogenized on ice initially with ~10 to 15 strokes followed by 30 strokes to extract nuclei. The nuclear pellets were obtained by centrifugation and resuspended in 6 mL homogenization buffer (10mM HEPES, pH 7.6, 25 mM KCl, 1 mM EDTA, 1 mM EGTA, 1 M sucrose, 10% glycerol, 0.15 mM spermine, and protease inhibitor cocktail, PIC1). The buffer containing nuclei was layered on top of a 3 mL layer of homogenization buffer, and centrifuged at 5000×g for 1 h in a Beckman SW41 rotor. The nuclear pellets were then re-suspended in 0.3 mL nuclear lysis buffer (50 mM Tris pH 7.6, 10 mM EDTA and 1% SDS), and diluted with 0.6 mL immunoprecipitation (IP) dilution buffer (0.01% SDS, 1.1% Triton ×100, 167 mM NaCl, 16.7 mM Tris pH 7.6, 1.2 mM EDTA). Then, 0.3 mL of the nuclear lysate was sonicated for 25-30 cycles in 30 seconds at 4° C using BioRuptor twin sonicator (Diagenode). Sonicated chromatin was further diluted to 1 mL using IP dilution buffer. TGFβ1 was conjugated overnight at 4° C to Dynabeads Protein G (Life Technologies) using PBS containing 0.5% BSA and gentle rotation. Then, the sonicated DNA (0.33 mL made up to 1 mL in IP dilution buffer or 10% of input DNA) was incubated overnight with TGFβ1 beads at 4° C with gentle rotation. The samples were then washed twice with wash buffer I (20 mM Tris, pH 7.4, 150 mM NaCl, 0.1% SDS, 1% Triton ×100, 2 mM EDTA), thrice with alternate wash buffer III (10 mM Tris, pH 7.4, 250 mM LiCl, 1% NP-40 alternate, 0.7% deoxycholate, 1 mM EDTA), twice with 0.2% Triton ×100 TE buffer, and twice with TE buffer containing 50 mM NaCl. The DNA was recovered from the beads by incubating samples at 65° C overnight in 0.3 M NaCl followed by Proteinase K digestion. The DNA was purified using QIAquick PCR purification kit (Qiagen) and subjected to real-time qPCR.

### Functional assay *in vivo*

Subcutaneous xenograft tumor model was adopted to assess the effects of Calycosin on tumorigenicity in nude mice. The procedures of animal experiments were done according to the Institutional Animal Care and Use Committee guidelines in Southern Medical University. 2×10^6^ T47D Cells were injected subcutaneously into the right flank for 4 weeks of male nude mice (Guangdong Animal Center, China). 7 weeks later, the tumors were isolated and weighted. The size of tumor was calculated as: V (mm^3^) = L×W^2^×0.5 (W: tumor short axis, L: tumor long axis). All subsequent procedures were performed using sterile technique.

### Statistical analysis

All experiments were repeated at least three times. Statistical analysis was performed using the IBM SPSS 21.0 statistical software. The data were represented as means ± standard deviation. The differences between groups were analyzed using analysis of variance (ANOVA) or Student's t-test. *P*<0.05 was considered statistically significant.
